# WEE1 Inhibitor: Clinical Development

**DOI:** 10.1007/s11912-021-01098-8

**Published:** 2021-07-16

**Authors:** Anthony Kong, Hisham Mehanna

**Affiliations:** 1 Institute of Head and Neck Studies (InHANSE), University of Birmingham, Birmingham, UK; 2 Comprehensive Cancer Centre, King’s College London, London, UK

**Keywords:** WEE1 inhibitor, Adavosertib, AZD1775, TP53 mutation, Biomarker, Clinical trials

## Abstract

**Purpose of Review:**

WEE1 inhibitor has been shown to potential chemotherapy or radiotherapy sensitivity in preclinical models, particularly in p53-mutated or deficient cancer cells although not exclusively. Here, we review the clinical development of WEE1 inhibitor in combination with chemotherapy or radiotherapy with concurrent chemotherapy as well as its combination with different novel agents.

**Recent Findings:**

Although several clinical trials have shown that WEE1 inhibitor can be safely combined with different chemotherapy agents as well as radiotherapy with concurrent chemotherapy, its clinical development has been hampered by the higher rate of grade 3 toxicities when added to standard treatments. A few clinical trials had also been conducted to test WEE1 inhibitor using TP53 mutation as a predictive biomarker. However, TP53 mutation has not been shown to be the most reliable predictive biomarker and the benefit of adding WEE1 inhibitor to chemotherapy has been modest, even in TP53 biomarker-driven studies.

**Summary:**

There are ongoing clinical trials testing WEE1 inhibitor with novel agents such as ATR and PAPR inhibitors as well as anti-PDL1 immunotherapy, which may better define the role of WEE1 inhibitor in the future if any of the novel treatment combination will show superior anti-tumor efficacy with a good safety profile compared to monotherapy and/or standard treatment.

## Introduction

WEE1 kinase is a serine-threonine kinase that regulates G2/M checkpoint transition [1–3]. WEE1 triggers G2/M arrest through inhibitory phosphorylation on Tyr15 of CDK1 (Cdc2) and preventing entry into mitosis to allow DNA repair during DNA damage [1–3]. WEE1 inhibition could result in high CDK1 activity and the cell progressing through the G2/M checkpoint without adequately repairing DNA damage, and thus generating mitotic catastrophe and cell death [1–3]. In addition, WEE1 inhibition also catalyzes the phosphorylation on CDK2 and leads to its high activity, resulting in aberrant DNA replication and DNA double-stranded breaks [1–3].

Since P53 is a key regulator of the G1/S checkpoint of cell cycle, TP53 mutations could impair its function resulting in the cells’ reliance on the later G2/M checkpoint [1–3]. Thus, WEE1 inhibition in the presence of TP53 mutation could disrupt both G1/S and G2/M, resulting in synthetic lethality and enhancing chemotherapy cytotoxity [1–3]. AZD1775 (MK1775), a potent, selective small molecule inhibitor, has been shown to potentiate the activity of various DNA-damaging chemotherapeutic agents such as gemcitabine, carboplatin, and cisplatin in p53-deficient cells and in vivo [4]. However, one report showed that WEE1 inhibition sensitized various cancer cell lines to antimetabolite chemotherapeutics regardless of p53 functionality [5].

Although most preclinical studies have demonstrated potent chemo-sensitizing activities when AZD1775 is combined with chemotherapy targeting S-phase including DNA cross-linking agents like platinum, nucleoside analogs or inhibitors of DNA metabolism, or topoisomerase poisons [4, 5], there is also preclinical evidence to support the combination of AZD1775 with anti-mitotic agents such as taxanes in several tumor models [6–9]. Being the pivotal negative regulator of Cdk1/Cyclin B1 activity, Wee1 kinase is required for normal entry into and exit from mitosis. It has been proposed that WEE1 inhibitor promotes cancer cells to prematurely enter mitosis as a result of bypassing the G2 cell-cycle checkpoint [6] as well as delays mitotic exit, resulting in mitotic arrest [7]. Importantly, WEE1 inhibitors are also antimitotic inhibitors by inducing mitotic arrest resulting in cell death regardless of cell-cycle phase prior to treatment. The addition of WEE1 inhibitor to paclitaxel increased total time in mitosis compared to either treatment alone in HELA cells and can sensitize breast cancer cells to paclitaxel treatment, providing a rationale of combining paclitaxel with WEE1 inhibitor [7]. It was also shown that AZD1775 significantly inhibited cell proliferation and induced apoptosis and cell cycle arrest in gastric cancer cells [8]. In addition, the combination of AZD1775 with paclitaxel was more effective than paclitaxel alone in gastric cancer cells and in gastric cancer orthotopic-transplanted mice models [8]. In another study, it was shown that Wee1 inhibition strengthened the spindle assembly checkpoint and extended mitosis to enhance the apoptotic cell death effect of antimicrotubule cancer drugs such as paclitaxel in several cancer cells and primary human adult lymphoblastic leukemia cells [9].

## Clinical Trials of Adavosertib Monotherapy and in Combination with Different Chemotherapies

In a phase 1 trial conducted in 25 patients with refractory solid tumors, the MTD monotherapy dose of adavosertib was determined to be 225 mg bd for 2.5 days given for 2 weeks out of a 3-week cycle (total dose = 2250mg every 3 weeks) (Table 1) [10]. The most common toxicities were myelosuppression (including anemia, neutropenia, and thrombocytopeni) and diarrhea. The dose-limiting toxicities (DLT) were supraventricular tachyarrhythmia (n=1) and myelosuppression (n=1) [10]. The study enrolled six patients with BRCA-mutant solid tumors at the MTD dose and two patients were confirmed to have a partial response (PR) [10].

**Table 1:** Adavosertib doses in monotherapy or in combination with different chemotherapies, radiotherapy, or immunotherapy in various clinical trials

Clinicaltrials.gov	Clinical study title	Adavosertib as monotherapy or in combination	Adavosertib doses
NCT01748825	Phase I study of single-agent AZD1775 (MK-1775), a wee1 kinase inhibitor in patients with refractory solid tumors [10]	Monotherapy	MTD dose 225 mg bd for 2.5 days given for 2 weeks out of a 3-week cycle (2250mg q21d)
NCT00648648	Phase I study evaluating wee1 inhibitor AZD1775 as monotherapy and in combination with gemcitabine, cisplatin, or carboplatin in patients with advanced solid tumors [11]	Part 1: Monotherapy Part 2: Combination with gemcitabine, carboplatin or cisplatin chemotherapy	Single doses of 325mg, 650mg and 1300mg 225 mg bd for 2.5 days week 1 with carboplatin AUC 5 D1 q21; 200 mg bd for 2.5 days week 1 with cisplatin 75mg/m^2^ q21d; 175 mg od for 2 days weeks 1–3 with gemcitabine 1000 mg/m^2^ weeks 1–3 (D1, D8 and D15) q28d
NCT02272790	Adavosertib with chemotherapy (CT) in patients with platinum-resistant ovarian cancer (PPROC): an open-label, four-arm, phase II study [12]	Combination with: 1) gemcitabine 2) paclitaxel 3) carboplatin 4) pegylated liposomal doxorubicin (PLD)	1) 175 mg od for 2 days weeks 1–3 with gemcitabine 800 mg/m^2^ weeks 1–3 (D1, D8 and D15) q28d 2) 225 mg bd for 2.5 days weeks 1–3 with weekly paclitaxel 80mg/m^2^ D1, 8 and 15 q28d 3) 225 mg bd for 2.5 days week 1 or weeks 1–3 with carboplatin AUC 5 D1 q21d 4) 175 mg bd for 2.5 days week 1 or 225 mg bd for 2.5 days week 1 with PLD 40mg/m^2^ D1 q28d
NCT02151292	A randomized double-blind placebo-controlled phase II trial comparing gemcitabine monotherapy to gemcitabine in combination with adavosertib in women with recurrent, platinum-resistant epithelial ovarian cancer [13]	Combination with gemcitabine	175 mg od for 2 days weeks 1–3 with gemcitabine 1000 mg/m^2^ weeks 1–3 D1, D8 and D15 q28d
NCT01164995	Phase II study of wee1 inhibitor AZD1775 plus carboplatin in patients with TP53-mutated ovarian cancer refractory or resistant to first-line therapy within 3 months [14]	Combination with carboplatin	225 mg bd for 2.5 days week 1 with carboplatin AUC 5 D1 q21
NCT01357161	A biomarker-enriched, randomized phase II trial of adavosertib (AZD1775) plus paclitaxel and carboplatin for women with platinum-sensitive TP53-mutant ovarian cancer [15••]	Combination with paclitaxel and carboplatin	225 mg bd for 2.5 days week 1 with paclitaxel 175mg/m^2^ D1 and carboplatin AUC 5 D1 q21
NCT02448329	Study of AZD1775 in combination with paclitaxel, in advanced gastric adenocarcinoma patients harboring TP53 mutation as a second-line chemotherapy	Combination with paclitaxel	225 mg bd for 2.5 days week 1 with weekly paclitaxel 80mg/m^2^ D1, 8, and 15 q28d
NCT02037230	Dose escalation trial of the wee1 inhibitor adavosertib (AZD1775) in combination with gemcitabine and radiation for patients with locally advanced pancreatic cancer [16•]	Combination with gemcitabine and radiation	RP2D 150mg od (D1–2, D8–9 q21d) with four cycles of gemcitabine (1000 mg/m^2^ D1 and 8 q21d) plus radiation (administered concurrently for cycles 2 and 3)
NCT02508246	A phase I clinical trial of AZD1775 in combination with neoadjuvant weekly docetaxel and cisplatin before definitive therapy in head and neck squamous cell carcinoma [17•]	Combination with docetaxel and cisplatin followed by definitive chemoradiation	MTD 150 mg bd for 2.5 days week 1 with weekly cisplatin 25mg/m^2^ and weekly docetaxel 35mg/m^2^ q21d
NCT02617277	Open-label, multi-center, phase I study to assess safety and tolerability of adavosertib plus durvalumab in patients with advanced solid tumors [18]	Combination with durvalumab	RP2D 150mg bd (3 days on, 4 days off; treatment D15–17, D22–24) with durvalumab 1500 mg (D1 q28d)

In another study, adavosertib was given as a single monotherapy dose (part 1) or in combination with different chemotherapies (gemcitabine, carboplatin, or cisplatin) (part 2) in patients with advanced solid tumors (Table 1) [11]. In part 1, single doses of 325mg, 650mg, and 1300mg were given and the most common drug-related adverse events (AEs) were diarrhea (22%) and fatigue (22%) but the MTD was not reached as a single dose [11]. In part 2 with the combination treatment, 19% had a serious treatment-related AEs including fatigue (58%), nausea (67%), vomiting (35%), diarrhea (41%), anemia (32%), neutropenia (32%), and thrombocytopenia (44%) [11]. The MTD doses for adavosertib were determined to be 225 mg bd for 2.5 days every 21 days in combination with carboplatin (AUC 5); 200 mg bd for 2.5 days every 21 days with cisplatin 75mg/m^2^; and 175 mg od for 2 days weekly for 3 consecutive weeks out of every 4-week cycle in combination with gemcitabine (1000 mg/m^2^ weekly for 3 consecutive weeks out of every 4-week cycle), respectively (Table 1) [11]. In the 176 evaluable patients treated with a combination of adavosertib with chemotherapy, 10% achieved PR. Further analysis was done on baseline tumor samples from 52 patients, of which four (21%) of the 19 patients with tumors harboring TP53 mutation achieved a PR while only four (12%) of the 33 patients with TP53 wild-type tumors achieved a PR [11].

One four-arm phase II study has assessed the efficacy and safety profile of adavosertib in combination with carboplatin, gemcitabine, weekly paclitaxel, or pegylated liposomal doxorubicin in 94 patients with platinum-resistant epithelial ovarian, fallopian tube, or primary peritoneal cancer (NCT02272790) (ASCO 2019) [12]. The objective response rate (ORR) and median progression-free survival (PFS) were 31.9% and 5.5 months respectively but the highest anti-tumor efficacy was seen in the carboplatin AUC5 with adavosertib cohort (225mg bd for 2.5 days week 1–3 D1–3, 8–10, and 15–17) (Table 1) with a response rate of 66.7% and median PFS of 12 months [12]. The biomarker study using NGS showed that TP53 was the most common genetic aberration but there was no clear correlation between genomic markers and clinical responses or specific tumor types. In this study, it was shown that all patients had at least one treatment-emergent adverse event (TEAE) with an incidence of grade 3 TEAE of 40.4% and grade 4 TEAE of 41.5% and TEAEs lead to discontinuation of adavosertib in 12.8% of patients. The most common ≥ grade 3 TEAE included anemia (33%), neutropenia (45.7%), thrombocytopenia (31%), vomiting, and diarrhea (both 10%). In view of the high ≥ grade 3 TEAE, the authors recommended further optimization of dosing, schedule, and supportive medications [12].

A double-blind randomized phase 2 trial showed that addition of adavosertib (175mg od on D1–2, D8–9, and D15–16) to gemcitabine (1000mg/m^2^ IV D1, D8, and D15 in a 28-day cycle) (Table 1) improved PFS from 3.0 to 4.6 months (HR 0.56 (95% CI 0.35–0.90, p=0.015 Log rank) compared to placebo arm in patients with recurrent platinum-resistant/refractory high-grade serous ovarian cancer (HGSOC) [13](NCT02151292). In addition, there was also a statistically significant improvement in overall survival (OS) from 7.2 to 11.5 months (HR 0.56 (95% CI 0.34–0.92, P=0.022) and increased number of patients with PR from 3 to 21% (p=0.02). Again, there was increased grade 3/4 AEs especially hematological toxicity including anemia (31 vs 18%), thrombocytopenia (31 vs 6%), and neutropenia (62 vs 30%) in adavosertib arm compared to placebo arm, respectively [13].

Most of the preclinical and clinical studies have been performed using WEE1 inhibitor from AstraZeneca, namely AZD1775 (MK1775 or adavosertib). Another highly selective and potent WEE1 inhibitor Debio0123 from Debiopharm is currently being tested with carboplatin in a first-in human phase 1 study (NCT03968653) and the first two dose levels were shown to be well tolerated with manageable toxicity (ESMO 2020).

## Biomarker Driver Studies

The clinical applications of TP53 mutations have been proven to be extremely complex since P53 function can be inactivated by various mechanisms, including somatic and germline mutations as well as polymorphisms [19, 20].

In the abovementioned phase I study of adavosertib monotherapy at MTD dose in adult patients with refractory solid tumors, confirmed PRs were observed in two patients with germline BRCA1 mutations but no responses were observed in five patients with TP53 mutations [10]. This study indicates that TP53 mutations may not be the best predictive biomarker and genetic aberrations in DNA repair pathways may also be important in predicting sensitivity to WEE1 inhibitor. However, this study did not specifically focus on tumors with TP53 mutations. There are now results available from a few studies specifically assessing the effects of adavosertib in tumors with TP53 mutations.

In a phase II study of adavosertib in combination with carboplatin in patients with p53 mutated ovarian cancer refractory or resistant (< 3 months) to standard first-line platinum-based therapy, patients were re-exposed to carboplatin and thus served as their own control [14]. Patients were treated with adavosertib 225 mg orally twice daily over 2.5 days with carboplatin (AUC5) every 21-day cycle (Table 1) until disease progression. Despite enrolling platinum refractory/resistant patients, there was encouraging antitumor activity with an overall response rate of 43%, which exceeded the response rates of 11 to 21% for other second-line single agents like paclitaxel, pegylated liposomal doxorubicin, bevacizumab, and topotecan [14]. The median progression-free and overall survivals were 5.3 months and 12.6 months, respectively [14]. The most frequent grade 3 or 4 AEs were hematological toxicities including anemia (9%), neutropenia (37%), and thrombocytopenia (48%). The proven TP53 mutation is a mandatory inclusion criterion and the majority of TP53 mutations were in exons 5 to 8. The exploratory genetic analysis using next generation sequencing (NGS) showed prolonged responses in two patients, one with Cyclin E and the other with BRCA1, MYC, and Cyclin E mutations, respectively [14].

In another double-blind randomized phase II trial study following an open-label safety run-in, women with TP53-mutated, platinum-sensitive ovarian cancer were randomized to either placebo or oral adavosertib (225 mg bd for 2.5 days/21-day cycle) plus carboplatin (AUC5) and paclitaxel (175 mg/m^2^) (Table 1) [15••]. The trial screened 394 patients, of which 121 were randomized and 273 patients were not excluded (with 259 patients were negative for TP53 mutation). The important to note for this trial is that the primary endpoint was volumetric progression-free survival by enhanced RECIST v1.1 (ePFS (volumetric)), which assesses changes in tumor volume and may allow earlier detection of tumor response and progression in contrast to the standard RECIST v1.1, which assesses tumor size changes in single dimension. The co-primary endpoint was safety and the PFS by RECIST v1.1 (single dimension) was secondary endpoint. In addition, the CA-125 level was included in the analysis of ORR according to Gynecologic Cancer InterGroup (GCIG) criteria [21] but not in the PFS analyses.

There was no difference in the complete response (CR) rate (11.9% in adavosertib arm vs 8.9% in placebo arm) and PR rate (62.7% in adavosertib arm vs 61.3% in placebo arm) using enhanced RECIST 1.1 and CA-125. Although the study met the pre-specified criterion (two-sided P < 0.2) for superiority of adavosertib over placebo on ePFS, the differences between the two arms were small (HR, 0.63; 95% CI, 0.38–1.06; P=0.080; median ePFS, 7.9 and 7.3 months for adavosertib and placebo, respectively). For the secondary endpoint, the difference in the median PFS assessed by RECIST 1.1 was greater, 9.9 months in adavosertib arm compared to 8.0 months for placebo (HR, 0.55; 95% CI, 0.32–0.95; nominal p= 0.030) [15••]. Ironically, by using ePFS as primary endpoint, they actually saw a smaller difference between the two groups. The study did not power to detect OS difference and did not see a difference between the two groups.

There was higher incidence of grade ≥3 AEs and SAEs with adavosertib arm (78% and 41%, respectively) compared to placebo arm (65% and 20%) with the most common grade ≥3 AEs reported being hematological toxicities following adavosertib treatment [15••]. The PFS was longer in adavosertib arm for all subtypes of TP53 mutation (including missense, truncation or splice-site mutation, hotspot mutation). The separation of PFS Kaplan-Meier curves between groups occurred relatively late suggested that maintenance adavosertib monotherapy could be of value after completion of 6 cycles of chemotherapy combination with adavosertib and warrant further investigation, especially if monotherapy could have decreased toxicity compared with that its combination with chemotherapy while maintaining efficacy.

The study also looked at specific gene defects assessed by NGS to identify predictive biomarker. They found long PFS (>274 days) for patients with CCNE1-amplified disease in adavosertib arm but unable to draw conclusions on the genes of interest (BRCA1, BRCA2, and CCNE1) due to too few patients with these mutations and heterogeneous mutational profiles [15••].

There is an ongoing study of adavosertib in combination with weekly paclitaxel in TP53 mutated gastric cancer patients (NCT02448329) with adavosertib 225 mg BID q 12 hours (× 5 doses) is given on days 1 to 3 with weekly paclitaxel 80mg/m^2^ administered on days 1, 8, and 15 of a 28-day cycle (total dose of AZD1775 = 1250mg every 4 weeks) (Table 1).

The above studies demonstrate that there remains conflicting evidence of predictive value of TP53 mutations in relation to WEE1 inhibitor, and further studies may be needed to study different TP53 inactivation mechanisms in relation to response to WEE1 inhibitor with different chemotherapies and tumor subtypes.

## Combination with radiotherapy and concurrent chemotherapy

The preclinical data has shown that the WEE1 inhibitor is a potential radiosensitizer and the addition of AZD1775 to irradiation significantly decreased clonogenic survival and increased apoptosis in cervical cancer cells as well as decreased tumor growth significantly more in the xenografts and the PDXs compared to radiotherapy alone [22]. Another study showed that AZD1775 sensitized esophageal cancer cells to radiotherapy in vitro and the combination treatment resulted in marked tumor regression of esophageal cancer mouse xenografts [23]. To date, there does not seem to have any completed or ongoing study from the combination of adavosertib with radiotherapy alone (without concurrent chemotherapy) although there have been several clinical studies combining adavosertib with radiotherapy and concurrent chemotherapy.

A dose escalation study of adavosertib (od on days 1, 2, 8, and 9 every 21 days) with four cycles of gemcitabine (1000 mg/m^2^ days 1 and 8 in 21 day cycle) plus radiation (administered concurrently for cycles 2 and 3) was conducted in patients with locally advanced pancreatic cancer (Table 1) [16•]. The recommended phase 2 dose (RP2D) was 150 mg/day for adavosertib [16•]. Eight patients (24%) out of 34 enrolled patients experienced a DLT including neutropenic sepsis/thrombocytopenia (n=1), abnormal liver function test (n=1), anorexia/nausea (n=3), fatigue (n = 2), abdominal pain (n = 1), and mental state disturbance (n = 1). The median PFS was 9.4 months and the median OS was 21.7 months, which is substantially higher than historical data of the combination of gemcitabine with radiation in similar patients [16•].

In another open-label phase I clinical trial for patients with borderline-resectable or unresectable stage III/IVB HNSCC suitable for definitive chemoradiation, increasing doses of adavosertib was administered orally bd over 2.5 days on week 1 followed by its combination with weekly cisplatin (25mg/m^2^) and docetaxel (35mg/m^2^) for three additional weeks (Table 1); the MTD for adavosertib was established at 150mg orally bd for 2.5 days with grade 3 diarrhea as the only DLT [17•]. There was promising anti-tumor efficacy in the 10 evaluable patients including 5 patients with PR, 4 patients with stable disease (SD), and one patient with progressive disease (PD) after week 2 and went on to received chemoradiation [17•]. Thus, this triplet combination of adavosertib, cisplatin, and docetaxel showed promising anti-tumor activity with good safety profile and warrants further investigation. It would be interesting to compare this treatment regimen with induction docetaxel, cisplatin, and 5FU (TPF) chemotherapy followed by chemoradiation, which is offered as standard treatment at many cancer centers.

We are waiting further studies combining adavosertib with chemoradiation to report their results soon, including one phase 1b study assessing the combination of adavosertib with radiotherapy and concurrent cisplatin chemotherapy in head and neck squamous cell carcinoma [24]. There may also be a role of assessing adavosertib as radiosensitizer when concurrent chemotherapy was deemed to be inappropriate due to renal impairment or co-morbidities.

## Novel Therapeutic Combination

Despite promising results seen on the combination of adavosertib with various chemotherapies or radiation with concurrent chemotherapy, the toxicities of adavosertib in combination with chemotherapy would likely to limit its future development. It is possible that adavosertib would have better safety and tolerability profiles as well as promising anti-tumor efficacy when combined with other non-chemotherapeutic novel agents.

The preclinical experiments have supported the combination of adavosertib with olaparib and/or ATR inhibitor for synergistic anti-tumor effect [25–30]. There are ongoing trials testing the combination of adavosertib with olaparib (NCT02511795) and/or ATR inhibitor (NCT03330847). There is also an interest in combining WEE1 inhibitor with immunotherapy. One phase I study has assessed the safety of adavosertib plus durvalumab in patients with advanced solid tumors (NCT02617277) [18]. The most common grade ≥3 AEs were fatigue (15%), nausea (9%), and diarrhea (11%) [18]. There were 2 DLTs, namely nausea (n = 2) and diarrhea (n = 1), and the recommended phase 2 dose for adavosertib was 150 mg bd (3 days on, 4 days off; treatment D15–17, D22–24) with durvalumab 1500 mg (D1 q28d). There was preliminary evidence of antitumor activity with a disease control rate for the overall cohort of 36% [18]. In view of the better safety profiles of the combination of adavosertib with immunotherapy compared with its combination with chemotherapies, further studies are warranted to assess its role in combination with immunotherapy in various tumors.

## Conclusions

Various preclinical and clinical studies have supported the use of WEE1 inhibitor to potentiate the activity of chemotherapies and/or radiotherapy in various tumors. However, the toxicities of the combination of adavosertib with chemotherapy and/or radiotherapy have limited its clinical development, especially when the additional efficacy results have been modest and have not translated into an overall survival benefit. In addition, there has not been a single reliable predictive biomarker for adavosertib monotherapy or in combination with other treatments. Various studies are now ongoing to test the novel combination of adavosertib with targeted therapies such as PARP and ATR inhibitors as well as immunotherapy. It is likely that these novel combinations may have better safety profiles as well as promising anti-tumor efficacies based on the previous preclinical findings. Despite the disappointment of clinical studies of adavosertib with chemotherapies and/or radiotherapy thus far, there remains a possible role of WEE1 inhibitor in combination with other targeted agents and/or immunotherapy, which may become clearer in the future.
